# Typhidot - A blessing or a menace

**DOI:** 10.12669/pjms.312.5934

**Published:** 2015

**Authors:** Khalid Mehmood, Ayesha Sundus, Iftikhar Haider Naqvi, Mohammad Faisal Ibrahim, Osama Siddique, Nida Faisal Ibrahim

**Affiliations:** 1 Dr. Khalid Mehmood, FRCP, Head of Department, Medical Unit 3, Civil Hospital Karachi, Pakistan; 2 Dr. Ayesha Sundus, MBBS, House Officer, Civil Hospital Karachi, Pakistan; 3 Dr. Iftikhar Haider Naqvi, FCPS, Assistant Professor, Medical Unit 3, Civil Hospital Karachi, Pakistan; 4 Dr. Mohammad Faisal Ibrahim, MBBS, Graduate Medical Student, Dow University of Health Sciences, Karachi, Pakistan; 5 Dr. Osama Siddique, MBBS, Graduate Medical Student, Dow University of Health Sciences, Karachi, Pakistan; 6 Dr. Nida Faisal Ibrahim, MBBS, Graduate Medical Student, Dow University of Health Sciences, Karachi, Pakistan

**Keywords:** Typhoid, Typhidot, Sensitivity, Specificity, Blood culture

## Abstract

**Objectives:**

Typhoid remain an increasing problem in Third world countries like Pakistan. A reliable, easy and affordable rapid diagnostic test is a need for our clinicians, many of whom consider Typhidot to be promising. Typhidot has been used as the only tool to diagnose typhoid fever by general practitioners and consultants despite its low sensitivity and specificity causing misdiagnosis and treatment. We therefore conducted this study to evaluate the sensitivity and specificity of Typhidot in patients with fever.

**Methods:**

A retrospective analysis of a total of 145 febrile patients was done. Blood culture and Typhidot along with other relevant investigations had been performed in each case. Sensitivity, specificity and the association of Typhidot to the diagnosis was found using SPSS v16.0.

**Results:**

Out of 145 patients, 15(10.3%) had positive blood culture for Salmonella typhi, 7 (4.8%) had positive culture for salmonella paratyphi and 94(64.8%) had positive culture for other organisms. Twenty nine (20%) patients had negative culture results. Forty seven (32.4%) patients had only IgM positive on Typhidot, 7(4.8%) had both IgM and IgG positive and 91(62.8%) had both IgM and IgG negative. Amongst the 130 patients with diseases other than typhoid, 50(38.5%) showed a positive Typhidot result. Amongst the 15 patients with typhoid, 11(73.3%) showed a negative Typhidot result. The sensitivity of Typhidot was found to be 26.7% and the specificity was 61.5%. The Positive Predictive Value (PPV) was 7.4% and the Negative Predictive Value (NPV) was 87.9%.

**Conclusion:**

Even though Typhidot is rapid, easy and affordable, its use should be discouraged due to low sensitivity and specificity and insignificant (p=0.067) association to the disease.

## INTRODUCTION

Typhoid fever continues to remain a menace worldwide especially in developing countries like Pakistan. In 2000, it was estimated that over 2.16 million episodes of typhoid occurred worldwide, resulting in 216,000 deaths, and that more than 90% of this morbidity and mortality occurred in Asia.^1^ Although improved water quality and sanitation constitute ultimate solutions to this problem, vaccination in high-risk areas is a potential control strategy recommended by WHO for the short-to-intermediate term.^2^

Although the causative bacterium was first described 132 years ago, most typhoid patients do not have access to reliable laboratory diagnosis, since the appropriate facilities and techniques usually still do not exist in economically poor areas of endemicity.^3^,^4^ It is important to design and evaluate sensitive and specific diagnostic tests for early detection and treatment of typhoid fever. A simple, reliable and affordable rapid diagnostic test has been a long felt need for developing countries like Pakistan. Isolation of Salmonella typhi on blood culture remains the best method of diagnosis but many other tests including TUBEX, Typhidot, Widal and others are widely used in developing countries for several reasons. Unfortunately, neither the Widal test, which remains in widespread use in the developing world, nor any of the other sero-diagnostic tests that have since been developed has proven sufficiently sensitive, specific, and practical to be of value in areas where this disease is endemic.^5^ They suffer from antibody persistence after cure or immunization especially in case of IgG, lack of determination of locally appropriate cutoffs, and cross-reactivity.^6^,^7^ Sensitivity is also an issue with PCR-based tests, due to the low venous blood bacterial concentration.^8^,^9^ Typhoid diagnosis in the tropics could be enhanced by making the identification of *S. typhi* in blood cultures simpler, less expensive, and faster.^10^

Typhidot is a simple test and requires no training of staff for specialized equipment. Hence it is widely used in all third world countries as a reliable and affordable means of detecting typhoid. Typhidot is done on a dot ELISA kit that detects IgM and IgG antibodies against the outer membrane protein (OMP) of the Salmonella typhi. The Typhidot test is expected to become positive within 2–3 days of infection. The test is based on the presence of specific IgM and IgG antibodies. IgM shows recent infection whereas IgG signifies remote infection. Typhidot was 67% sensitive and 54% specific, with 85% positive and 81% NPVs.^11^ In a study in Egypt in which Typhidot was compared with a new ELISA not yet commercially available, the sensitivity and specificity were again very low.^12^

This study thus aimsed on evaluating the sensitivity and specificity of the widely used Typhidot keeping blood culture as a gold standard as a diagnostic tool for this grave problem.

## METHODS

### Patients

A retrospective study was done on patients from Civil Hospital Karachi (2000 bed hospital catering patients from Karachi, Interior Sindh and parts of Balochistan) which is affiliated with Dow University of Health Sciences and Anklesaria Nursing Home Karachi. Patients presented with a history of fever of at least 3 days. Whatever their ultimate diagnosis might have been, their complete blood count, Typhidot, Blood Culture and other relevant investigations (chest x-ray, urine d/r, urine c/s, ultrasound, dengue serology, stool culture, sputum culture, MRI brain) were arranged.

**Figure:**
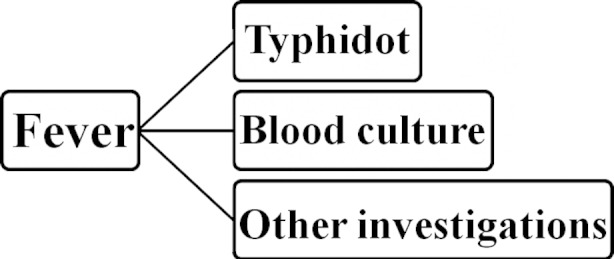


### Approval

The study was approved by Ethical Review Board of Dow University of Health Sciences, Karachi, Pakistan.

### Typhidot

TYPHIDOT Rapid IgG/IgM is an indirect solid phase immunochromatgraphic assay designed for the qualitative detection and differentiation of specific IgM and IgG antibodies against specific Salmonella typhi antigen in human whole blood, serum or plasma. Around 40 micro liter of blood was collected and inserted in the well of the test cassette. If the blood contains specific antibodies for Salmonella typhi then a color change is observed. This test is based on the principle of antigen-antibody complexes formed with the conjugated antigens present. Antibodies and reagents for capture of anti-S. typhi IgM and IgG are immobilized onto cellulose nitrate membrane as test lines. When the test sample is added to the sample pad, it migrates upwards together with dye conjugated to S. typhi antigens.

### Blood Culture

Blood samples were inoculated in thioglycolate broth and incubated at 37°C for 7 days. Positive blood cultures were further processed, colonies were identified using standard biochemical tests and antibiotic susceptibility was checked by Kirby - Bauer disc diffusion methods.^13^

### Analysis

Analyses were done on SPSS version 16. Fischer exact test for association was used to check the association of both Typhidot and blood culture with typhoid fever since both the data were unfit for chi squared test. Considering blood culture as a gold standard, the sensitivity and specificity of Typhidot was found. Patients who showed positive on both Typhidot and blood culture were considered ‘true positives’ and paratyphoid and other diseases as ‘true negatives’.

## RESULTS

One hundred and forty five patients gave consent to participate in the study. Out of these 59 (40.7%) were males and 86 (59.3%) females. The mean age of the patients was 30.50±19.966 years.

Blood counts were done as a routine baseline and one hundred and thirteen (77.9%) patients had a low hemoglobin level while 32(22.1%) had normal hemoglobin.

One hundred and eighteen (81.4%) patients had a normal total leukocyte count, 17(11.7%) had a high count and 10(6.9%) had a low count. Platelet Count was normal in 100(69.0%) patients, high in 19(13.1%) and low in 26(17.9%).

Forty seven (32.4%) patients had only IgM positive on Typhidot, 7(4.8%) had both IgM and IgG positive and 91(62.8%) had both IgM and IgG negative. (Table-I)

**Table-I:** Typhidot results.

IgM positive	47(32.4%)
Both IgM and IgG positive	7(4.8%)
Both IgM and IgG negative	91(62.8%)
True positives	4
False negatives	11
False positives	50
True negatives	80
Sensitivity of Typhidot	26.7%
Specificity of Typhidot	61.5%
Positive Predictive Value of Typhidot	7.4%
Negative Predictive Value of Typhidot	87.9%
Association with typhoid fever	P=0.067

Out of 145 patients, 15(10.3%) had positive culture for Salmonella typhi, 7 (4.8%) had positive culture for salmonella paratyphi and 94(64.8%) had positive culture for other organisms. Twenty nine (20%) patients had negative culture results. (Table-II)

**Table-II:** Blood Culture results.

Positive for typhi	15 (10.3%)
Positive for paratyphi	7 (4.8%)
Positive for other organisms	94 (64.8%)
Association with typhoid fever	P<0.0001

Out of 15 (10.3%) patients who had typhoid, true positives (patients who had typhoid proven on blood culture and positive Typhidot) were 4 and false negatives (patients who had typhoid proven on blood culture and gave negative Typhidot) were 11.

Out of 130 (89.7%) patients who had other diseases (including paratyphoid), false positives (patients who had other diseases and gave Typhidot positive) were 50 and true negatives (patients who did not have typhoid and gave negative Typhidot) were 80. The other diseases found are presented in Table-III.

**Table-III:** Diseases in which typhidot was found positive.

Final Diagnosis	Number of patients	Based On:
Typhoid	4 (7.4%)	S. typhi on blood culture
Paratyphoid	4 (7.4%)	S. paratyphi on Blood culture
Urinary Tract infections	5 (9.3%)	Urine D/R ad urine culture
Liver Abscess	5 (9.3%)	Ultrasound Abdomen
Malaria	10 (18.5%)	Malarial parasite positive on malarial immunochromaographic test
Respiratory Tract Infection	3 (5.6%)	Sputum culture
Pulmonary Tuberculosis	2 (3.7%)	Chest X-ray Findings
Dengue	7 (13.0%)	Dengue IgM IgG positive
Gastroenteritis	6 (11.1%)	Stool culture
Brain Tuberculosis	1 (1.9%)	MRI brain
Rheumatoid Arthritis	2 (3.7%)	Rheumatoid factor positive
Tonsillitis	3 (5.6%)	Examination Findings
Sinusitis	2 (3.7%)	Examination findings
Total	54	

The sensitivity of Typhidot was found to be 26.7% and the specificity was 61.5%. The Positive Predictive Value (PPV) of Typhidot was 7.4% and the Negative Predictive Value (NPV) of Typhidot was 87.9%.

The Fischer exact test of association showed Typhidot to be insignificant for diagnosis of typhoid fever (p=0.067). It also displayed blood culture to be highly significant in the diagnosis of typhoid fever (p<0.0001).

## DISCUSSION

We evaluated Typhidot results versus blood culture results for the patients with acute febrile illness of three or more days at Civil Hospital Karachi. Overall Typhidot did not provide as promising results as clinicians expect it to. It displayed very low sensitivity and specificity despite being rapid and easy to perform.

The hospital participating in this study is a 2000 bed tertiary care centre catering a wide variety of patients from all over Karachi and many other parts of the province of Sindh. Patients belonged to all age groups, gender, ethnicities and diseases.

In our evaluation, the sensitivity of Typhidot was extremely low (26.7%) and so was the specificity (61.5%) compared to 67% and 54% in a previous study in Bangladesh.^4^ The PPV was 7.4% and NPV was 87.9% compared to 85% positive and 81% NPVs according to a research in Bangladesh.^11^ Even though Typhidot detects IgM during the acute phase and IgG later, it displayed very low positives in the blood culture positive individuals (4 out of 15)and many positives (50 out of 80) in other bacteremic infections and diseases. (Table-III) Specificity of Typhidot-M in another similar study in India was 37.5%.^14^ With such low specificities, clinicians need to be cautioned not to have too much dependence on Typhidot as an early diagnostic tool since it could lead to erroneous management of the patients’ disease. Neither TUBEX^®^ nor Typhidot^®^ was both sensitive and specific in two evaluations undertaken in Viet Nam.^15^,^16^ Moreover, the World Health Organization (WHO) has issued no recommendations on the use of typhoid rapid antibody tests either.^2^ Accurate diagnosis is required for fruitful management of patients and also to find incidence and prevalence of typhoid in low resource setting like ours. And for this purpose the use of tools like Typhidot need to be used with cautiously.

The time elapsed between the onset of symptoms and serum collection can affect the performance of antibody-based tests.^17^ We did not consider this aspect in our study and all patients were tested as soon as they presented. This could be a reason for the variable findings of the test in various typhoid patients.

Cross reactivity between antigens of other diseases that gave positive Typhidot and outer membrane protein of S. typhi could be another reason for the low specificity of Typhidot. It has been observed in previous studies that bacteraemia due to *Non Typhoid Salmonella* may result in false-positive results with TUBEX^®^ because they have an O9 antigen in common with S. typhi.^18^

It is important to notify clinicians of these drawbacks of the widely used Typhidot so that adequate management of the patients is undertaken. since our study clearly demonstrates insignificant (p=0.067) association of Typhidot to the diagnosis of typhoid and to be positive in several other diseases including Urinary Tract Infection, liver abcess, malaria, respiratory tract infection, pulmonary tuberculosis, dengue fever, gastroenteritis, systemic lupus erythmatosus, viral arthritis, tonsillitis and sinusitis.

Ideally the Widal, TUBEX and other rapid diagnostic tests should also have been done on these patients to evaluate superior diagnostics. This can be analyzed in a further study.

It is concluded that the value of Typhidot test in diagnosing typhoid fever in a tertiary care centre in Karachi is very low so its use may be discouraged and instead use of blood culture should be increased for reliable results which is still the gold standard for diagnosis of typhoid and para typhoid fever. It is further recommended to do this study on a wider scale after excluding all compounding variables which may cause false positive Typhidot results.
